# Tracheotomy in Croup and Diphtheria

**Published:** 1875-12

**Authors:** 


Tracheotomy in Croup and Diphtheria.—Prof. Spence, Surgeon in Ordinary to the Queen in Scotland, in discussing the surgery of the air-passages, in his recent address in surgery before the

British Medical Association, (British Medical Journal, Aug. 14, 1875), said: "I have now performed tracheotomy for simple croup one hundred and three times, and saved thirty-four out of that number, or an average of about one life saved in three cases; and it must be remembered that at first I only operated as a last resort, and even yet I do not see my way to operate quite so early as some French Surgeons seem to do. I think, however, that there should be no delay when the character of the breathing and the contracted state of the thoracic parietes show that the lungs are not being distended with air. By operating early we avoid the risk of œdema or congestion of the lungs, and of the effects of non-oxygenized blood circulating in the brain.

"I think it right, however, to warn my young brethren that it will require some effort to bear up against discouraging results. I know of no class of cases in which the experience is so painful: an average gives little idea of it. You may have five or six cases in succession, all proving fatal, before you meet with one redeeming success; but then you have the temporary relief almost invariably afforded to the little sufferer; the resuscitations in some cases apparently dead; and, if you persevere, the average of success will come. Above all, we must recollect that, however disagreeable or unpleasant the operation may be to ourselves, we are bound to lose sight of that, and give the patient the only chance for life.

"In speaking of operations in croup, I have used the term simple and diphtheritic croup; and I have done so advisedly, because, whilst the average results of my operations have been as good in one disease as in the other, I consider them as essentially different diseases, and I do not believe that an extended experience would give the same amount of success in diphtheritic croup as in simple croup. It has been with no small amazement I have read some of the views recently propagated, that croup and diphtheritic croup are identical. I can hardly conceive two diseases more different, whether we consider them in the causation, symptoms, or sequelæ."

Prof. Spence drew attention to the difference many years ago in a paper read before the Edinburgh Medico-Chirurgical Society, and subsequently published; and, looking back on his large experience in croup and diphtheritic croup, he thinks the distinctive characters are too marked to allow him to consider the diseases as identical, merely because they possess one feature in common.

